# Donor-Site Morbidity and Quality of Life after Autologous Breast Reconstruction with PAP versus TMG Flap

**DOI:** 10.3390/curroncol29080448

**Published:** 2022-08-11

**Authors:** Angela Augustin, Petra Pülzl, Evi M. Morandi, Selina Winkelmann, Ines Schoberleitner, Christine Brunner, Magdalena Ritter, Thomas Bauer, Tanja Wachter, Dolores Wolfram

**Affiliations:** 1 Department of Plastic, Reconstructive and Aesthetic Surgery, Medical University of Innsbruck, Anichstrasse 35, 6020 Innsbruck, Austria; 2 Department of Obstetrics and Gynecology, Medical University of Innsbruck, Anichstrasse 35, 6020 Innsbruck, Austria

**Keywords:** breast cancer, mastectomy, autologous breast reconstruction, PAP flap, TMG flap, donor-site morbidity, quality of life

## Abstract

The transverse myocutaneous gracilis (TMG) and the profunda artery perforator (PAP) flap are both safe choices for autologous breast reconstruction originating from the same donor region in the upper thigh. We aimed to compare the post-operative outcome regarding donor-site morbidity and quality of life. We included 18 patients who had undergone autologous breast reconstruction with a PAP flap (n = 27 flaps). Prospective evaluation of donor-site morbidity was performed by applying the same questionnaire that had already been established in a previous study evaluating TMG flap (n = 25 flaps) outcome, and results were compared. Comparison of the two patient groups showed equivalent results concerning patient-reported visibility of the donor-site scar and thigh symmetry. Still, the TMG group was significantly more satisfied with the scar (*p* = 0.015) and its position (*p* = 0.001). No difference was found regarding the ability to sit for prolonged periods. Donor-site wound complications were seen more frequently in the PAP group (29.6%) than in the TMG group (4.0%). Both groups expressed rather high satisfaction with their quality of life. Both flaps show minimal functional donor-site morbidity and high patient satisfaction. To minimize wound healing problems in PAP patients, thorough planning of the skin paddle is necessary.

## 1. Introduction

Breast cancer is the most common cancer in women worldwide, affecting approximately 12.9% of all women in industrialized countries [1]. Due to the improved survival rates of breast cancer over the past decades and the increasing trend in the use of bilateral and contralateral prophylactic mastectomy, aesthetic demands and life quality claims come to the fore [2,3,4]. Furthermore, the percentage of patients seeking breast reconstruction after mastectomy is increasing [5]. Autologous breast reconstruction is still the primary method used for permanent restoration of the breast mound following any type of mastectomy due to its natural aesthetic result and high patient satisfaction [6]. Several studies using patient-reported outcome measures found a higher quality of life and greater satisfaction after autologous breast reconstruction compared to implant-based techniques [7,8,9]. Abdominal-based flaps, more precisely the deep inferior epigastric perforator (DIEP) flap, is considered the gold standard for breast reconstruction [10,11,12]. However, alternative flaps must be considered in patients who do not offer sufficient abdominal volume, who underwent previous abdominal surgery, or even liposuction, potentially compromising the perforating vessels, or in patients who refuse an abdominal scar [12]. Free flaps from the thigh have proven to be a reliable alternative, herein the transverse myocutaneous gracilis (TMG) flap has long been favored due to its inconspicuous scar and low long-term donor-site morbidity [13,14,15]. The TMG is easier and faster to harvest than any perforator flap. However, a major disadvantage, aside from the shorter pedicle, is volume loss due to muscle degeneration over time and the possible need for additional lipofilling procedures [16,17,18]. With further surgical refinements concerning the use of perforator flaps, the profunda artery perforator (PAP) flap has emerged as an alternative approach [19]. It profits from a longer pedicle, higher flap weight and lower donor-site complication rates [16,20]. Although much literature has been published to date concerning flap harvesting techniques, versatility of the flap as a single or stacked flap, preoperative perforator mapping and post-operative complications, only limited data can be found concerning patient satisfaction with the reconstruction and donor-site outcomes.

The latest advancements in the surgical treatment of breast cancer, permitting near complete preservation of the breast skin envelope, enable improved aesthetic outcomes after breast reconstruction. In this work, we set a strong focus on patient satisfaction as well as patient-reported outcomes after NSME or SSME and thigh flap-based breast reconstruction. We aimed to compare patient-reported contentment with the type of reconstruction, donor-site morbidity as well as quality of life in patients with TMG versus PAP autologous breast reconstruction.

## 2. Materials and Methods

### 2.1. Study Design

This single-center study was approved by the Institutional Ethics Committee of MEDICAL UNIVERSITY INNSBRUCK (protocol code 1058/2020, 28 October 2020). Informed consent for photo documentation, the operation and anonymized evaluation and publication of data was obtained in written form from all patients. We included a total of 18 patients (27 PAP flaps). Inclusion criteria were defined as age > 18 years, breast cancer diagnosis, high-risk genetic disposition or recurrent infections of the breast, uni- or bilateral breast reconstruction and post-operative course longer than 12 months. We excluded patients with metastatic disease, severe psychiatric disorders and follow-up less than 12 months. Patient demographics including age, body mass index (BMI), smoking habits and comorbidities as well as post-operative complications were documented retrospectively.

Prospective evaluation of the patient-reported outcome with regard to donor-site morbidity was conducted using a clinically established standard post-operative questionnaire at our department. Evaluation of the TMG group was published by Pülzl et al. in 2011 [15], we now conducted an equivalent data collection in the PAP group and compared our results to the historic cohort. Patients were asked about complaints resulting from elevation of the flap, their satisfaction with the results, their quality of life and sexuality. Patients were able to choose from five possible responses to the questions: “no”, “a little”, “fair”, “much” and “most”. In accordance with the previous study, cosmetic evaluation of the donor-site concerning thigh symmetry, appearance of the scars and shape of the thighs was conducted by two senior plastic surgeons based on standardized pre- and post-operative photo documentation.

### 2.2. Patients

**Figure 1:**
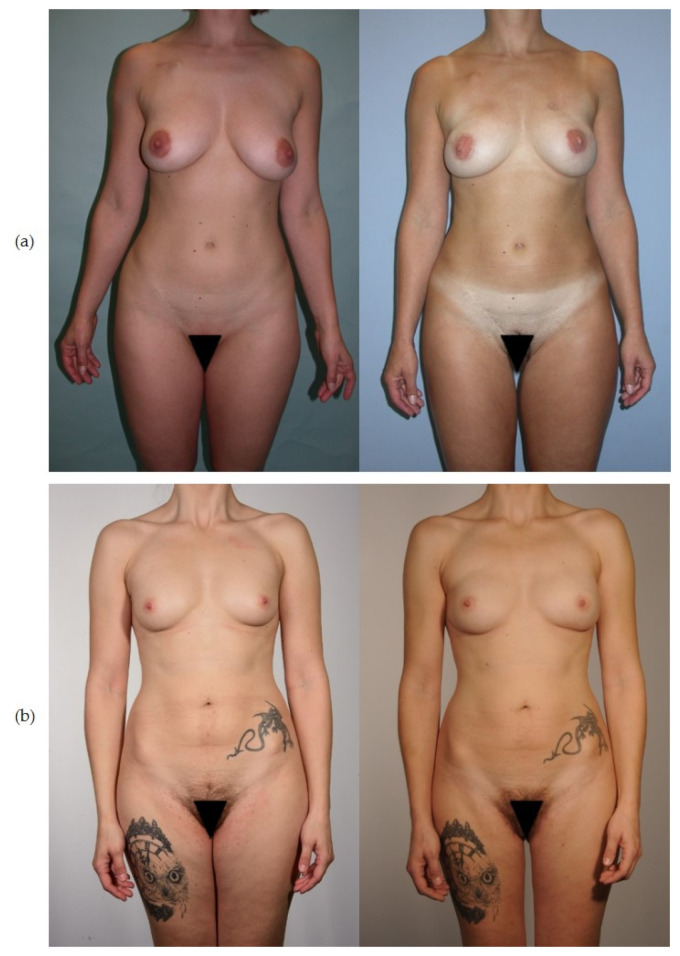
(Left) Preoperative and (right) post-operative views of bilateral breast reconstruction with donor-site in the thigh region: (**a**) TMG flap and (**b**) PAP flap.

From January 2016 to November 2019, a total of 29 patients underwent uni- or bilateral breast reconstruction with a PAP flap at our department. Six patients were excluded due to metastatic disease or severe psychiatric disorder, three patients were lost to follow-up due to relocation. Twenty patients with a post-op follow-up of at least 12 months were invited for clinical examination, of whom 18 patients consented to participate and nine of those underwent bilateral PAP flap breast reconstruction. Consequently, 27 flaps and 27 donor-sites were evaluated (Figure 1b). In the historic TMG cohort 25 flaps and donor-sites in 22 patients had been evaluated with a mean post-operative follow-up of 10 months (Figure 1a).

### 2.3. Statistical Analysis

Statistical analysis was performed using ©Microsoft Excel 2016 (Microsoft Corporation; https://office.microsoft.com/excel access on 12 November 2019) and ©MedCalc Statistical Software Ltd. (Comparison of means calculator. https://www.medcalc.org/calc/comparison_of_means.php Version 20.027 access on 30 July 2021). For statistical analysis, the possible responses on the questionnaire were evaluated according to a scoring system as follows: 1 point was given for the response “no” and 5 points for “most”. Comparison between the two patient groups (PAP and TMG) was achieved with an independent samples *t*-test. Data are presented as mean ± standard deviation along with range, where applicable; *p* < 0.05 was considered statistically significant.

## 3. Results

### 3.1. Patient Characteristics

Evaluation of the 18 included patients following autologous PAP flap breast reconstruction showed a mean age of 43.6 (±7.4) years at the time of surgery, average BMI was 21.6 (±2.3) kg/m² (range 17.9–27.5 kg/m²) (Table 1). Mean follow-up time was 34 (±15.8) months. Nine women underwent bilateral reconstruction, with 27 flaps and donor-sites being evaluated. Indication for mastectomy was diagnosed breast cancer in 63.0% (17/27), high-risk genetic disposition in 33.3% (9/27) and recurrent infection in 3.7% (1/27) of the flaps. Autologous breast reconstruction was performed in 85.2% (23/27 breasts) by primary intention in a single-stage surgery with the mastectomy, and in 14.8% (4/27 breasts) of the study patients as secondary reconstruction following an initial implant-based breast reconstruction.

Evaluation of complications showed in 29.6% (8/27) of operated thighs events classified as Grade 3a or 3b according to the Clavien–Dindo classification (hematoma, seroma, wound dehiscence, wound infection) necessitating operative revision [21]. We observed one (3.7%) free flap loss due to venous failure, altogether, post-operative complications concerning the breast added up to 22.2 % (6/27). Staged corrections due to aesthetic deficits or complaints in the field of surgery were considered as secondary revisions. Such corrections of the donor-site were performed in 11.1% (3/27) of thighs (lipofilling, neuroma resection and scar correction). Altogether, in 34.6% (9/26) secondary corrections of the breast were performed after reconstruction with a PAP flap, among them, consecutive lipofilling was performed in 26.9% (7/26) of the reconstructed breasts and two patients had a scar correction. Corresponding evaluations in the TMG group are presented in Table 2.

### 3.2. Questionnaire Evaluation

Table 3 shows all questions and the distribution of the chosen responses. Results from our previous study evaluating the TMG flap donor-site are likewise added. Questions 11, 13, 25 and 29 were answered in free text; the results are given in the text below. For Question 30, no *t*-test was performed because only two patients in the TMG group responded.

#### 3.2.1. Questions Regarding Donor-Site

**Figure 2:**
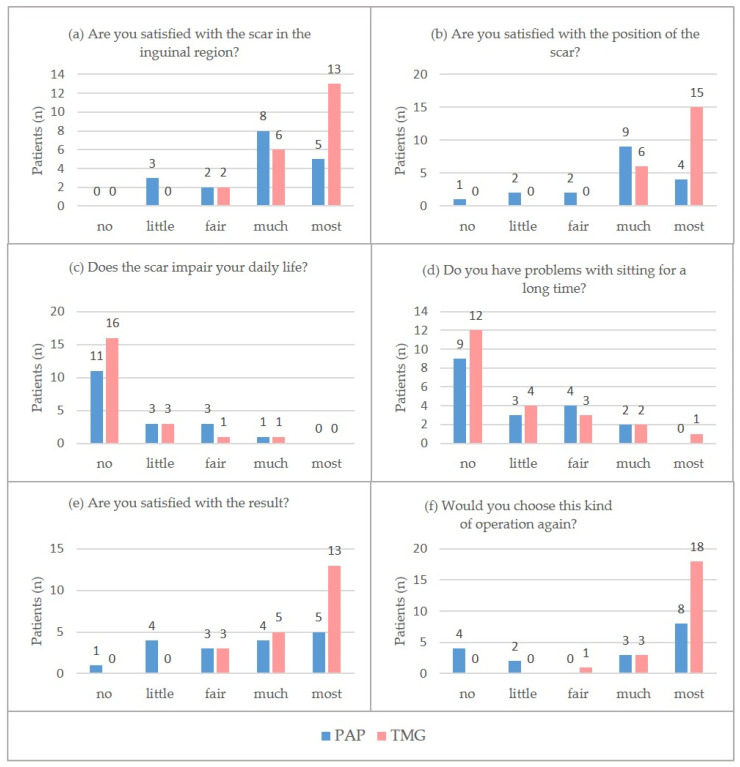
(**a**–**f**). Questions about the donor-site and breast reconstruction, “no answer” excluded.

Satisfaction with the scar in the inguinal region was in both groups rather positively rated, with “much” being the leading response in the PAP group (44.4%) and “most” in the TMG group (59.1%). Still, the TMG group was significantly more satisfied with the scar (*p* = 0.015) and its position (*p* = 0.001) (Figure 2a,b). Comparison of the two patient groups showed equivalent results concerning patient-reported visibility of the donor-site scar (Table 3; Questions 3–7) and thigh symmetry (Table 3; Questions 8–10). PAP patients significantly more often reported wound-healing problems than did TMG patients (2.56 ± 1.37 points versus 1.41 ± 0.94 points; *p* = 0.004). Questions 14–30 (Table 3) asked about scar-evoked afflictions. The PAP group reported a higher prevalence (*p* = 0.006) of sensory deficits (3.29 ± 1.32 points) than did the TMG group (2.09 ± 1.24 points). Sensitivity to cold and pressure were rated similarly, with both groups choosing “no” as the leading response (Table 3; Questions 16–18). No difference was found concerning the ability to sit for prolonged periods, with the majority of patients in both groups choosing “no” (50.5% in the PAP and 54.5% in the TMG group) or “a little” (16.7% in the PAP and 18.2% in the TMG group) (Figure 2d). In the PAP group six patients complained of being restricted in their leisure activities such as bike-riding, stretching, hiking and climbing. When asked about their willingness to choose this kind of operation again, 61.1% of the patients in the PAP group chose the responses “much” and most”, while 95.5% of the patients in the TMG group chose one of these options (*p* = 0.002; Table 3 and Figure 2f).

#### 3.2.2. Questions Regarding Breast Reconstruction

PAP and TMG patients both chose “most” as the leading response when asked about their satisfaction with the breast reconstruction result (Figure 2e). Still, distribution over the response options was greater even in the PAP group and therefore the ranking result is significantly lower than in the TMG group (3.47 ± 1.29 points versus 4.48 ± 0.73 points; *p* = 0.004).

#### 3.2.3. Questions Regarding General Condition

Both groups expressed rather high satisfaction with their quality of life (Figure 2c). With 4.28 ± 1.04 points in the PAP group and 4.77 ± 0.42 points in the TMG group a trend towards higher satisfaction in the TMG group was seen, although this was not statistically significant (*p* = 0.05).

#### 3.2.4. Questions Regarding Sexuality

Reported satisfaction with sexuality was higher in the TMG group (Table 3, Questions 42–46), but no difference was found in the questions addressing the correlation with reconstructive surgery (Table 3, Questions 47–50), where in both groups “little” to “fair” change in sexuality after the operation was reported.

### 3.3. Cosmetic Results

**Figure 3:**
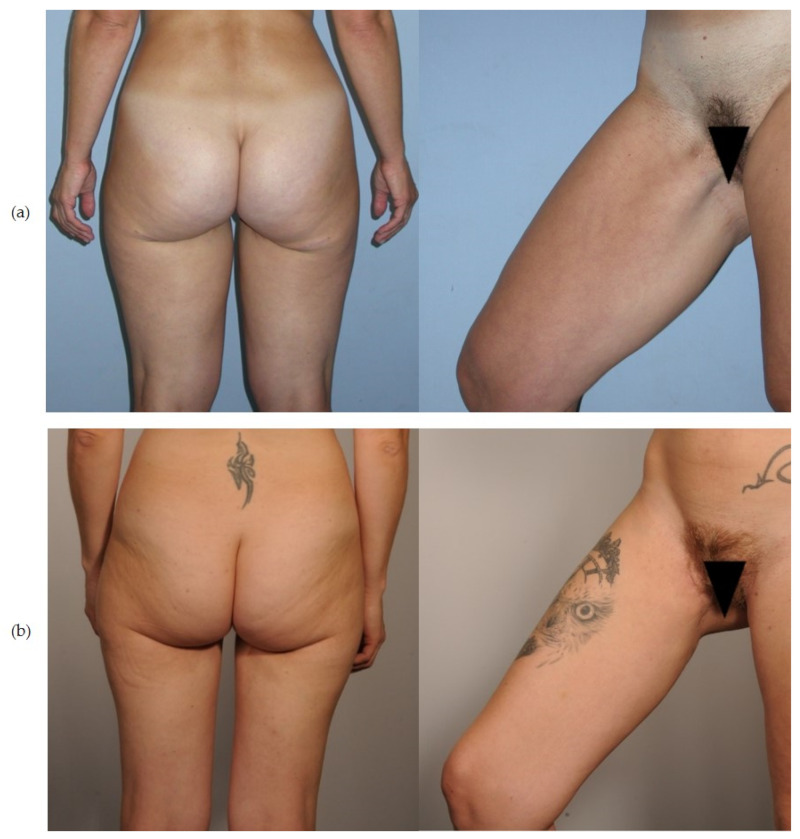
Cosmetic results. Post-operative views of donor-site after bilateral flap harvesting: (**a**) TMG flap and (**b**) PAP flap.

Measurement of the circumference of the thigh revealed in PAP patients with unilateral flap harvesting a difference of 3.28 (± 1.31) cm, while PAP patients with bilateral donor-sites presented a difference of 0.75 (± 0.66) cm between the two legs (Figure 3; below). Table 4 shows the difference in thigh circumference in patients with unilateral flap harvesting for both groups. Mean difference between both thighs was 0.96 cm greater in PAP patients compared to the TMG group.

Evaluation of thigh symmetry and appearance of the scars based on standardized photo documentation showed better results in PAP patients, while thigh shape was rated better in TMG patients (Figure 3).

## 4. Discussion

An enhanced treatment regime for breast cancer patients must provide a rehabilitation of the psychological burden and aesthetic defect due to mastectomy [22,23]. Several groups have reported that both the TMG and the PAP flap are safe choices for autologous breast reconstruction, with tissue from the thigh achieving good aesthetic results especially in small- to medium-sized breasts [24,25,26,27,28,29]. The advantages of the PAP flap as compared to the TMG muscle flap are the longer pedicle length and greater flap volume. Jo describes in his systematic review of the two flaps an average pedicle length of 9.4 cm and an average flap weight of 342 g for the PAP and 6.4 cm and 316.2 g for the TMG flap [16]. Moreover, the PAP includes only skin and fat tissue and therefore matches the paradigm “replace like with like” ideally. Perforator preparation prevents the need for muscle sacrifice. On the contrary, the TMG flap is anatomically reliable, easier and overall faster to harvest. With advances in skills and knowledge of perforator flap preparation, the PAP flap has almost completely displaced the TMG flap for autologous breast reconstruction with tissue from the thigh at our department.

Donor-site complications must be well considered when evaluating methods of breast reconstruction, especially since flap reconstruction showed a higher rate of surgical complications compared to implant-based reconstruction after mastectomy in general [30]. Surgical site complications for the TMG and the PAP flap are well described in previous studies, and review articles show comparable incidences of wound dehiscence, seroma and hematoma formation with other donor-sites for autologous breast reconstruction [16,31,32]. Despite muscle harvesting in TMG patients, functional deficits in the leg are rare [20]. The most frequent functional impairments after TMG harvesting are seen to be sensory deficits [32]. Haddock reports a return to normal lower extremity musculoskeletal conditions by six months after PAP harvesting, while Craggs reports that all patients were able to return to their normal activities of daily living after TMG harvesting [17,29]. A comparison of patient-reported outcome for both techniques is not available in the literature so far.

In our study, satisfaction with the scar and its position was higher in the TMG group, although results concerning the restrictions posed by the scar in daily life, and also the ability to sit for prolonged periods, were comparable to those seen in the PAP group. Comparison of the two patient groups also showed no difference concerning patient-reported visibility of the donor-site scar. We hypothesize that those results might be linked to the higher rate of donor-site complications in the PAP group (29.6% vs. 4.0% in the TMG group). Memories of complications and negative emotions connected to a protracted healing period may give rise to a more critical evaluation of the scar, analogous to Craggs‘ finding of a significant correlation between complications and lower patient satisfaction with the TMG donor-site [17].

Research in the literature reveals varying donor-site complication rates. Jo concludes in a review article that the TMG flap has a higher pooled estimate of wound dehiscence (9.73% for TMG and 7.59% for PAP), but recent studies addressing the PAP flap showed higher rates of donor-site complications [16]. Haddock, Cho and Fosseprez describe a cumulative rate of complications (seroma, hematoma, wound dehiscence) of 13.9%, 17.5% and 35.3%, respectively [29,33,34]. Modification of the flap design as an “extended” or “Fleur-de-Lys” PAP were suggested in order to increase flap volume and allow reconstruction in large-sized breasts; donor-site wound dehiscence with this technique is reported in 14–23.9% of thighs [35,36]. We used the Clavien–Dindo classification for evaluation of post-operative complications and events classified as grade 3a or 3b were considered. Therefore, minor circumstances treated under local anesthesia were also counted and this might also contribute to the revealed high rate of complications.

We considered that the increased complication rate in our PAP patients is associated with a rather low BMI (21.6 ± 2.3 kg/m²; range 17.9–27.5 kg/m²) in our cohort. In these patients only limited tissue is available in the thigh. Therefore, wide undermining is often required, and wound margins are rather thinly trimmed to harvest sufficient flap volume. Distal preparation and undermining is required in all PAP patients since the perforator for the flap is often approximately 2 cm distal to the vascular pedicle of the gracilis muscle [37,38]. Another reason for wound complications may be the missing mobilization of the thigh soft tissues after harvesting the PAP flap. When harvesting the TMG flap, the whole gracilis muscle is included up to its tendon at knee level, thus leading to wide mobilization of the lower wound margins. This leads to better distribution of tension in the donor-site and, hence, might be a reason for fewer healing problems. The technically demanding method of PAP flap harvesting certainly necessitates a learning curve, therefore we did not include all patients with a PAP flap breast reconstruction at our department but started evaluation only from 2016 when we had already gained one year of experience. During the early stage of PAP flap reconstructions, high rates of donor-site complications turned out to be the biggest challenge. We observed decreasing incidences of donor-site wound healing problems with technical adaptations and cautious planning of the skin paddle, which we now limit to 6 cm width. For surgical planning, all our PAP patients underwent computed tomography angiography (CTA) of the donor-site for perforator mapping preoperatively. Interestingly, there was no correlation between lower satisfaction with the donor-site and the need for secondary corrections since the TMG group had touch-up procedures in 36.0% of the thighs compared to 11.1% in the PAP group. Moreover, in the reconstructed breasts secondary corrections were more frequent in TMG flaps than in PAP flaps (40.0% and 34.6%, respectively). research in the literature revealed even higher rates (54.1–61.0%) of secondary lipofilling in patients with a TMG flap [17,18]. Volume loss in the breast after autologous reconstruction has been described by Wilting but secondary tissue atrophy of the denervated gracilis muscle in TMG flaps may even lead to an increased need for secondary volume augmentation of the breast in these patients [39]. Furthermore, the rather low BMI (21.6 ± 2.3 kg/m²; range 17.9–27.5 kg/m²) in the PAP group may explain a volume deficit in the reconstructed breasts due to the limited tissue available in the donor region.

Sexual satisfaction was lower in the PAP group, but patients did not deem the reconstructive surgery to be responsible for this. One possible explanation for this fact might be that the evaluation of the PAP patients was performed between January 2020 and May 2021, thus coinciding with the COVID-19 pandemic. Yuksel reports significantly decreased quality of sexual life during the pandemic, which may be applicable to our patients as well [40].

The present study has some limitations to report. Pülzl et al. published the previous study in 2011, while reconstructive surgery in the included TMG patients was undertaken from 2002 to 2004. There is a significant time leap to the evolving autologous reconstructions with PAP flaps, which were performed from 2016 to 2019. The patient groups were also operated by different surgeons, as two senior surgeons performed the TMG reconstructions and another three senior surgeons did the PAP reconstructions. Due to our study design, including a historic cohort, we were obliged to use the same questionnaire again to keep the groups comparable for evaluation, therefore we could not revert to a validated form. 

## 5. Conclusions

Both TMG and PAP flaps show minimal functional donor-site morbidity but a trend towards higher patient satisfaction after TMG reconstruction was found. However, compared to TMG, the PAP donor-site reveals wound healing disadvantages. Our strategy to minimize wound healing problems in PAP patients is to restrict the skin paddle width to 6 cm. Still, risk of wound dehiscence should be discussed openly with patients preoperatively to allow informed consent. We recently switched to offering patients a PAP flap with a vertical skin paddle and comparison of the patient-reported outcome for these two different techniques used to plan the skin paddle will be the subject of a further study.

## Figures and Tables

**Table 1:** Patient characteristics PAP group.

Characteristic	Mean	(std)
Age (years) *	43.6	(7.4)
Follow-up (months) *	34.0	(15.8)
BMI (kg/m²) *	21.6	(2.3)
Flap volume (cc) †	327.7	(108.2)
	*n*	(%)
Active smoker *	1	(5.6)
Time of reconstruction †		
Primary	23	(85.2)
Secondary	4	(14.8)
Indication for mastectomy †		
Breast cancer	17	(63.0)
Prophylactic	9	(33.3)
Mastitis	1	(3.7)
Positive genetic testing *	4	(22.2)
Radiotherapy *		
Previous radiotherapy	3	(16.7)
Adjuvant	7	(38.9)
No	8	(44.4)
Chemotherapy *		
Previous chemotherapy	7	(38.9)
Adjuvant	4	(22.2)
No	7	(38.9)

100%: * *n* = 18 patients, † *n* = 27 flaps.

**Table 2:** Post-operative complications and secondary corrections.

Characteristic	PAP *n* *	(%)	TMG *n* †	(%)
Complications breast	6	(22.2)	11	(44.0)
Complications thigh	8	(29.6)	1	(4.0)
Secondary corrections breast	9 ˜	(34.6)	10	(40.0)
Secondary corrections thigh	3	(11.1)	9	(36.0)

100%: * *n* = 27 PAP flaps, ˜ *n* = 26 PAP flaps (1 flap loss), † *n* = 25 TMG flaps.

**Table 3:** Questionnaire.

	**PAP**	**TMG**	**PAP**	**TMG**	**PAP**	**TMG**	**PAP**	**TMG**	**PAP**	**TMG**	**PAP**	**TMG**	**PAP**	**TMG**	**T Test**
**Questions**	**No** **1 pt.**	**No** **1 pt.**	**Little** **2 pts.**	**Little** **2 pts.**	**Fair** **3 pts.**	**Fair** **3 pts.**	**Much** **4 pts.**	**Much** **4 pts.**	**Most** **5 pts.**	**Most** **5 pts.**	**No Answer**	**No Answer**	**Points** **(Mean)**	**Points** **(Mean)**	***p*** **-Value**
Donor-site															
1. Are you satisfied with the scar in the inguinal region?	0	0	3	0	2	2	8	6	5	13	0	1	3.83	4.52	0.015 *
2. Are you satisfied with the position of the scar?	1	0	2	0	2	0	9	6	4	15	0	1	3.72	4.71	0.001 *
3. Is the scar visible?	3	6	7	6	5	5	3	4	0	0	0	1	2.44	2.33	0.741
4. Is the scar optically disturbing to you?	6	12	8	6	3	2	1	1	0	0	0	1	1.94	1.62	0.246
5. Is the scar visible in front?	11	12	5	6	1	1	1	2	0	0	0	1	1.56	1.67	0.703
6. Is the scar visible from behind?	3	7	7	5	3	2	4	4	1	2	0	2	2.61	2.45	0.705
7. Is the scar red?	5	10	9	6	0	1	2	2	1	2	1	1	2.12	2.05	0.864
8. Have you noticed a difference in thigh circumference?	6	7	7	8	2	3	1	0	1	3	1	1	2.06	2.24	0.655
9. Are your thighs symmetrical?	3	2	1	2	2	1	7	11	5	6	0	0	3.56	3.77	0.610
10. If not, is the difference aesthetically disturbing to you?	5	9	2	5	3	1	1	0	1	1	6	6	2.25	1.69	0.216
11. How long was the healing period?															
12. Did you have problems with healing?	5	17	3	3	4	1	2	0	2	1	2	0	2.56	1.41	0.004 *
13. If yes, what problems?															
14. Do you have a loss of sensation around the scar?	1	9	5	7	4	3	2	1	5	2	1	0	3.29	2.09	0.006 *
15. If yes, is that disturbing to you?	6	6	5	2	2	2	3	2	0	1	2	9	2.13	2.23	0.830
16. Is the scar sensitive to pressure?	8	15	6	1	2	4	1	1	0	1	1	0	1.76	1.73	0.930
17. Is the scar sensitive to cold?	12	18	4	2	0	0	0	1	1	1	1	0	1.47	1.41	0.855
18. Is the scar painful?	9	18	5	0	3	3	0	0	0	1	1	0	1.65	1.45	0.506
19. Does the scar impair your daily life?	11	16	3	3	3	1	1	1	0	0	0	1	1.67	1.38	0.302
20. … for example, during swimming?	6	10	6	6	2	2	2	1	1	2	1	1	2.18	2.00	0.659
21. Do you have limitations in finding the right underwear / swimsuit caused by the scar?	8	16	3	2	4	1	1	1	2	2	0	0	2.22	1.68	0.206
22. Do you have limitations in movement caused by the scar?	8	13	7	6	3	1	0	1	0	1	0	0	1.72	1.68	0.893
23. Do you have problems with sitting for a long time?	9	12	3	4	4	3	2	2	0	1	0	0	1.94	1.91	0.935
24. Are there any things you can’t do because of the scar?	12	18	3	2	0	2	2	0	0	0	1	0	1.53	1.27	0.318
25. What things?															
26. Have you recognized swelling of the leg after the operation?	13	18	3	2	2	1	0	0	0	0	0	1	1.39	1.19	0.298
27. Do you have a heavy or flexed feeling in your leg?	10	15	5	4	1	2	2	0	0	1	0	0	1.72	1.55	0.592
28. Have you recognized changes in the genital region?	16	20	1	2	1	0	0	0	0	0	0	0	1.17	1.09	0.531
29. If yes, what has changed?															
30. If yes, are the changes disturbing to you?	8	0	1	2	1	0	0	0	0	0	8	20	1.30	2.00	na
31. Would you choose this kind of operation again?	4	0	2	0	0	1	3	3	8	18	1	0	3.53	4.77	0.002 *
*Breast reconstruction*															
32. Are you satisfied with the result?	1	0	4	0	3	3	4	5	5	13	1	1	3.47	4.48	0.004 *
33. Does the new breast meet your expectations?	2	1	2	1	2	3	8	7	3	10	1	0	3.47	4.09	0.104
34. Does the new breast harmonize with the other? †	2	2	1	1	1	2	3	5	2	7	0	2	3.27	3.89	0.196
*General condition*															
35. Are you able to work (including housework)?	1	0	0	0	0	1	3	3	14	18	0	0	4.61	4.77	0.503
36. Do you enjoy your life?	1	0	0	0	1	1	5	4	11	17	0	0	4.39	4.73	0.181
37. Do you accept your illness?	1	0	0	0	1	0	7	6	8	16	1	0	4.24	4.73	0.047 *
38. Do you sleep well?	1	1	2	1	2	6	5	2	8	12	0	0	3.94	4.05	0.775
39. Can you enjoy things that usually make you happy?	1	0	0	0	0	0	7	4	10	18	0	0	4.39	4.82	0.060
40. Are you satisfied with the quality of your life?	1	0	0	0	2	0	5	5	10	17	0	0	4.28	4.77	0.050
41. Do you think that a secondary breast reconstruction would have been better?	16	19	0	2	1	1	0	0	0	0	1	0	1.12	1.18	0.702
*Sexuality*															
42. Do you find yourself attractive?	1	0	1	0	6	5	8	13	0	2	2	2	3.31	3.85	0.029 *
43. Are you satisfied with your sexual condition?	1	0	1	0	3	3	9	12	1	5	3	2	3.53	4.10	0.041 *
44. Are you satisfied with the frequency of your sexual contacts?	1	0	0	0	5	5	8	7	1	8	3	2	3.53	4.15	0.036 *
45. Are you satisfied with the frequency of body contact from your partner?	0	0	0	0	4	2	4	5	7	12	3	3	4.20	4.53	0.212
46. Are you satisfied with your sexual reactions?	2	0	3	0	2	4	6	9	2	6	3	3	3.20	4.11	0.013 *
47. Did your sexuality change after the operation?	4	6	5	8	4	1	1	6	1	0	3	1	2.33	2.33	1.000
48. If yes, do you suffer from that?	5	7	3	7	2	1	2	2	0	0	6	5	2.08	1.88	0.609
49. Would you therefore suggest another kind of breast reconstruction to other patients?	12	18	0	1	2	1	2	0	0	0	2	2	1.63	1.15	0.090
50. Have you lost interest or enjoyment in sex caused by the donor-site?	10	13	1	5	4	3	0	0	0	0	3	1	1.60	1.52	0.768

* *p* < 0.05, † only patients with unilateral flap harvesting (PAP *n* = 9; TMG *n* = 19).

**Table 4:** Thigh circumference difference (cm) in patients with unilateral flap harvesting.

Flap	Mean	(std)
PAP *	3.28	(1.31)
TMG †	2.32	(1.69)

* *n* = 9, † *n* = 19 patients.

## Data Availability

The data presented in this study are available on request from the corresponding author.
